# MICROBIOTA COULD REDUCE THE BRACHIAL-ANKLE PULSE WAVE VELOCITY TO PROTECT FROM CARDIOVASCULAR DISEASE

**DOI:** 10.1093/geroni/igae098.2463

**Published:** 2024-12-31

**Authors:** Shengyin Zeng, Yaxin Li, Bangwei Chen, Lei Ruan, Jianguo Zhang, Shida Zhu, Cuntai Zhang, Tao Li

**Affiliations:** BGI Genomics, Shenzhen, Guangdong, China (People’s Republic); BGI Genomics, Shenzhen, Guangdong, China (People’s Republic); BGI Genomics, Shenzhen, Guangdong, China (People’s Republic); Tongji Hospital, Tongji Medical College, Huazhong University of Science and Technology, Wuhan, Hubei, China (People’s Republic); BGI Genomics, Shenzhen, Guangdong, China (People’s Republic); BGI Genomics, Shenzhen, Guangdong, China (People’s Republic); Tongji Hospital, Tongji Medical College, Huazhong University of Science and Technology, Wuhan, Hubei, China (People’s Republic); BGI Genomics, Shenzhen, Guangdong, China (People’s Republic)

## Abstract

Brachial-ankle pulse wave velocity (baPWV) is a risk factor for cardiovascular disease (CVD), and is associated with blood pressure and lipoprotein. However, research on the microbiota associated with baPWV has been limited. Here, we used two-sample bidirectional mendelian randomization (MR) analysis to assess the causality between gut microbiotas and baPWV. The exposure instrumental variables (IVs) were selected as significant independent variants (Linkage Disequilibrium (LD) R2 < 0.01; P-value < 5×10-8) from 1,521 gut microbiota genome-wide association studies (GWAS) summary data including class to species in the IEU OpenGWAS project. We performed baPWV GWAS analysis in Tongji-BGI research cardiovascular health (TORCH) cohort to obtain baPWV-related variants (rs141045630, rs11138617, rs77947040 and etc.) and selected suggestive variants (LD R2< 0.01; P-value < 1×10-5) as outcome IVs. The inverse variance weighted (IVW) was used as the primary MR analysis. In addition, we adjusted sex, age and other Framingham risk score factors. The sensitivity analysis involved changing the covariates, altering MR method and heterogeneity test. The analysis results showed higher abundance of Bifidobacterium adolescentis (P-value:1.81×10-4) and Lachnospiraceae (P-value:2.88×10-5) associated with lower baPWV. Moreover, MR evidence suggested a causal relationship between higher abundance of 3 microbiotas (Lactobacillales, P-value:1.72×10-3;Citrobacter, P-value:1.46×10-2; Ruminococcus, P-value:1.36×10-4) and higher baPWV. On the other hand, baPWV didn`t have potential causal effects on the abundance of B. adolescentis and Lachnospiraceae. In conclusion, a higher abundance of butyric acid-producing bacteria decreases baPWV and reduces arterial stiffness. We are currently performing more analyses with the metabolic pathway to explore more details in these results.

